# Cloning and Characterization of the Novel Endoglucanase Identified in Deep Subsurface Thermal Well of Biragzang (North Ossetia) by Metagenomic Analysis

**DOI:** 10.3390/biom15121710

**Published:** 2025-12-07

**Authors:** Natalia V. Trachtmann, Stepan V. Toshchakov, Anna O. Izotova, Aleksei A. Korzhenkov, Martha A. Evteeva, Gennady S. Kachmazov, Esperant E. Agboigba, Shamil Z. Validov

**Affiliations:** 1 Federal Research Center, Kazan Scientific Center of Russian Academy of Science, ul. Lobachevskogo, 2/31, Kazan 420111, Russia; n.trahtman@knc.ru (N.V.T.); e.agboigba@knc.ru (E.E.A.); 2 National Research Center, Kurchatov Institute, Ac. Kurchatov Square, 1, Moscow 123098, Russia; toschakov_sv@rrcki.ru (S.V.T.); izotova_ao@nrcki.ru (A.O.I.); korzhenkov_aa@nrcki.ru (A.A.K.); evteeva_ma@nrcki.ru (M.A.E.); 3 Faculty of Chemistry, Biology and Biotechnology, North Ossetian State University Named After K. L. Khetagurov, Vatutina Str., 44-46, Vladikavkaz 362025, Russia; kgssogutfi@yandex.ru

**Keywords:** cellulase Cel7465, metagenome, cloning, expression, thermophilic

## Abstract

The phylum *Armatimonadota* represents one of the least characterized bacterial lineages, with only three formally described species despite its widespread distribution in various environments. Deep subsurface thermal environments harbor significant microbial diversity and represent promising sources for novel enzyme discovery through metagenomic approaches. This study reports the identification, cloning, and biochemical characterization of Cel7465, a novel endoglucanase from an uncultured GBS-DC family within the order *Fimbriimonadales*. The enzyme was identified through metagenomic analysis of microbial mats from the Biragzang deep thermal well (North Ossetia, Russia, 48 °C) and successfully expressed in cells of *Escherichia coli* strain ArcticExpress (DE3). Phylogenetic analysis assigned Cel7465 to glycoside hydrolase family 5 subfamily 36. The purified recombinant enzyme exhibited optimal activity at 55 °C and pH 8.0, with high specific activity of 4347 U/mg. The enzyme demonstrated broad pH tolerance (50% activity retained from pH 4.0 to 10.0) and notable thermal stability, retaining 60% activity after one hour at 80 °C and 20% after four hours. Remarkably, the presence of Mn^2+^ ions enhanced enzyme activity more than 7-fold, while Mg^2+^ and Ca^2+^ ions provided moderate activation. Cel7465 represents the first biochemically characterized glycoside hydrolase from the order *Fimbriimonadales*, expanding our understanding of enzymatic capabilities within the understudied phylum *Armatimonadota* and demonstrating the continued potential of extreme environments as sources of novel industrial biocatalysts.

## 1. Introduction

Currently, a wide range of enzymes are employed as catalysts in numerous industrial processes, including the food, chemical, and biofuel industries. One of the most prominent industrial enzymes is cellulase, which catalyzes the breakdown of cellulose into simpler compounds. Cellulose is composed of D-glucose units that are linked by β-1,4-glucoside bonds and can serve as a source for the production of soluble carbohydrates. Endoglucanases (EC 3.2.1.4) are enzymes that hydrolyze internal β-1,4-glycosidic bonds within cellulose and related β-glucans, resulting in the formation of shorter oligosaccharides such as cellooligosaccharides or cellobiose. Together with exoglucanases (EC 3.2.1.91) and β-glucosidases (EC 3.2.1.21), they constitute the cellulolytic enzyme complex responsible for the depolymerization of cellulose into glucose units [1]. In contrast to exoglucanases, endoglucanases act randomly along the cellulose chain, making them essential initiators of cellulose degradation and key players in biomass conversion processes [2]. These enzymes are widely used in industry and research, and their diverse properties make them suitable for various applications. The extensive use of cellulose-acting enzymes has led to their rapid expansion in the global market.

Bacteria represent a prolific source of endoglucanases, as many species have evolved to degrade complex polysaccharides in both natural and extreme environments. However, the identification of novel bacterial endoglucanases remains challenging due to the cultivation difficulties of many potential producers and the limited functional characterization of enzymes from uncultured microorganisms [3]. Metagenomic approaches provide a powerful alternative, enabling the discovery of genes encoding glycoside hydrolases directly from environmental DNA without the need for microbial cultivation [4]. In recent decades, metagenomics has led to the discovery of a considerable number of bacterial glycoside hydrolases from diverse environmental sources. These include the fecal secretions of *Equus burchelli* [5], a metagenome from a biogas reactor powered by pig manure and rice straw [6], the *Vibrio* sp. G21 strain [7], and many others. Some of the discovered enzymes exhibited remarkable properties like acid resistance, thermostability and high catalytic activity, especially valuable for widespread use in biotechnological applications.

Current research focuses on mining metagenomes from extreme environments, where microorganisms experience physicochemical stresses that promote the evolution of robust and efficient hydrolases [8,9,10,11]. Using this approach, several cases of the successful identification and characterization of novel enzymes from uncultured microorganisms have been reported. For example, a novel glycoside hydrolase family 12 cellulase, Cel12E, was identified in a hydrothermal vent metagenomic library (originating from an uncultured hyperthermophilic archaeon) and demonstrated high thermostability and activity towards β-1,4-linked polysaccharides [12]. Another example is a GH16 laminarinase from the Jermuk hot spring (Armenia), cloned from environmental DNA, and experimentally characterized through recombinant expression and substrate hydrolysis tests [13]. However, despite the fact that metagenomic studies have enormous potential for the identification of novel gene families from uncultivated microorganisms [14], the number of experimentally confirmed GHs from truly uncultured taxa remains limited. The majority of functional assignments are predictions based on computational methods rather than direct biochemical validation [15].

In this study, we present the successful cloning, heterologous expression, and catalytic characterization of a novel endoglucanase from an uncultured representative of the *Fimbriimonadales* order. This enzyme was identified during metagenomic analysis of the microbial mats formed at the outlet of the deep Biragzang thermal well in the Republic of North Ossetia-Alania, Russia.

## 2. Materials and Methods

### 2.1. Bacterial Strains and Plasmids

All strains and plasmids used and constructed in this work are listed in Table 1.

### 2.2. Metagenomic Analysis of the Biragzang Thermal Well

#### 2.2.1. Sampling Procedure

The Biragzang deep thermal well is located on the right bank of the Ardon River in North Ossetia, approximately 35 km west of Vladikavkaz (coordinates: 42.9957° N, 44.2290° E, elevation: 685 m). This deep geothermal borehole, reaching 2370 m depth, discharges slightly alkaline (pH = 8.8) mineral water with a temperature of 48 °C and a mildly reducing redox potential (−20 to 0 mV). The outlet area is characterized by dense microbial mats that form beneath the water discharge, reflecting active microbial colonization. Sampling of water and microbial mats was performed using sterile consumables. Water (5–10 L) was passed sequentially through 0.45 µm and 0.22 µm Sterivex™ filters (Merck Millipore, Burlington, MA, USA) and stabilized in RNAlater (Thermo Fischer Scientific). Microbial mats and sediments were collected in 5-mL Eppendorf tubes. Excess liquid was removed, and RNAlater was added for preservation.

#### 2.2.2. Extraction of Environmental DNA and Library Preparation and Sequencing

DNA was extracted from filter membranes and sediment samples using the PowerLyzer PowerSoil kit (Qiagen, Hilden, Germany), following the manufacturer’s instructions. DNA samples were quantified using Qubit fluorometer (Thermo Fischer Scientific). For library preparation 50 ng of environmental DNA was fragmented using Covaris M220 sonicator (Covaris, Woburn, MA, USA) to the median fragment size of 550 bp. The libraries for Illumina sequencing were prepared with NebNext Ultra II DNA library preparation kit (New England Biolabs, Ipswich, MA, USA) according to the manufacturer’s instructions. Libraries were sequenced using Novaseq 6000 system (Illumina, San Diego, MA, USA) with 2 × 250 paired-end sequencing chemistry. Approximately 15 M of reads were obtained for all samples.

#### 2.2.3. Bioinformatic Analysis: Identification of Novel Cellulase Gene in the Thermal Well Metagenome and Its Phylogenetic Analysis

Sequencing reads from all samples were filtered and trimmed using *fastp* package and then co-assembled using SPAdes 3.15.0 in the metagenomic mode. Genecalling and in silico translation was performed using MetaGeneMark [18]. Search for cellulase genes was performed using reference HMM (https://www.ebi.ac.uk/interpro/entry/pfam/PF00150/logo/ accessed on 21 June 2025) by hmmsearch [19] using 1 × 10^−6^ e-value. For the reliable identification and phylogenetic analysis of cel7465 host, metagenomic contigs were binned with MetaWRAP pipeline [20]. The quality of obtained bins was checked with checkm2 program [21] and taxonomic assignment was performed with GTDB-toolkit v.2.1.0 using GTDB r207 database [22,23]. The analysis of bin abundances was performed with CoverM software v.0.7.0 software [22].

The GBS-DC-related bin was annotated with NCBI PGAP pipeline [24]. The search for glycoside hydrolases in in silico proteome was performed with dbCAN3 web-server [25]. The CAZY database was utilized to obtain protein sequences to construct a phylogenetic tree from biochemically characterized glycoside hydrolases of family 5 [26]. For each GH5 subfamily, three to four protein sequences with a unique Uniprot number were selected, preferably obtained from cultivated microorganisms. The alignment was constructed using the Mafft package with the mafft-linsi algorithm [27]. The alignment was trimmed using the TrimAl package in -automated1 mode [28]. The tree was constructed using IQ-TREE 3 with automatic model selection [29]. To augments the annotation of the tree, data on enzymatic activity in the form of EC Numbers obtained from the CAZy database and data on the cultivation temperature of the microbial source of the enzyme from the BacDive database were utilized [30]. The tree was visualized using iTOL v5 web-server [31].

### 2.3. Cloning and Expression of the Cellulase Gene (cel7465)

All basic DNA manipulation and transformation of microorganisms were performed according to standard protocols [32] and to the instructions provided by the manufacturers of the kits for genetic experiments. The candidate GH45gene identified in sample 4129B was amplified from environmental DNA by PCR using high-fidelity Q5 DNA polymerase (New England Biolabs) with phosphorylated primers (cel7465-F: 5′-ATGACCCAACGCACGCTGCC-3′ and cel7465-R: 5′-CTACATCGCCTGCAGCAGGTCTAAC-3′) previously treated with T4 polynucleotide kinase. The PCR products were analyzed by agarose gel electrophoresis and purified using the Qiaquick Gel Extraction Kit (Qiagen), following the manufacturer’s protocol. The purified amplicons were then cloned into the *Hinc*II site of the pUC19 plasmid vector via blunt-end ligation using T4 DNA ligase (New England Biolabs). Competent *E. coli* TOP10 cells (Thermo Fischer Scientific) were transformed with the ligation mixtures according to the supplier’s recommendations. Plasmid DNAs from positive colonies were isolated and verified by Sanger sequencing (ABI Prism 3730xl, Applied Biosystems, USA). Genetic map and sequence of the resulting plasmid (pUC19-cel7465) are given in Figure S1A.

The open reading frame was then amplified using primers cel7465-F-Bsp19I (5′-TTTTTTCCATGGCTACCCAACGCACGCTGCCGG-3′) and cel7465-R-Sfr274I (5′-TTTTCTCGAGCATCGCCTGCAGCAGGTCTAACATC-3′) with PhantaMax polymerase (Vazyme, Nanjing, China) from plasmid pUC19-cel7465 (Table 1). Sites for the recognition of *Bsp*19I and *Sfr*274I enzymes (isoshizomers of *Nco*I and *Xho*I, respectively) (SibEnzyme, Novosibirsk, Russia) were introduced into the primers, to allow for the further cloning of the gene into the pET28a vector at corresponding restriction sites. The obtained PCR fragments were hydrolyzed using *Bsp*19I and *Sfr*274I (SibEnzyme) and ligated into the pET28a vector, digested with the same restriction enzymes. So, the cloned cellulase gene was fused to a sequence encoding 6-His tag insuring presence of six histidine residues on C-terminus of the resulting protein. Plasmid DNAs were isolated from clones grown on selective medium after transformation of the DH5α strain with the ligation mixture and verified by sequencing (Evrogen, Moscow, Russia). Genetic map and sequence of the resulting plasmid (pET28a-cel7465) are given in Figure S1B. For the expression of Cel7465, two different strains of *E. coli* were used: ArcticExpress (DE3) and BL21 (DE3) pLys (Table 1). Both strains were transformed with the recombinant plasmid pET28a-cel7465 (Table 1). Expression of the protein was carried out in LB medium containing 50 µg/mL of kanamycin, and additionally, 25 µg/mL of chloramphenicol for the BL21(DE3) pLys strain and 10 µg/mL of gentamicin for the ArcticExpress (DE3) was added. Expression was induced at the middle of the logarithmic growth phase, with an optical density of 0.6–0.8 (OD_600_), using 0.5 mM β-D-1-thiogalactopyranoside (IPTG). The cells were incubated at different temperatures: 18 °C for the BL21(DE3) pLys strain for 16 h, and 12 °C for ArcticExpress (DE3), for 24 h. After incubation, the cells were harvested and centrifuged at 5000× *g* for further analysis. Protein expression was confirmed using 12% SDS-PAGE.

### 2.4. Protein Purification by Metal Affinity Chromatography (IMAC) and Size Exclusion Chromatography

The cell biomass was suspended in 50 mM Tris-HCl, 300 mM NaCl, 5% glycerol, 10 mM imidazole, and 5 µM β-mercaptoethanol at pH 7.2, supplemented with a protease inhibitor cocktail and phenylmethylsulfonyl fluoride. The mixture was disrupted by ultrasonication. To clear the cell lysate was centrifuged for 45 min at 50,000× *g* at 4 °C. The 6His-tagged cellulase was purified from the cleared cell extract using Ni-NTA metal affinity chromatography at 4 °C. The purity of the protein was analyzed on a 12% SDS-PAGE gel using a running buffer containing 25 mM Tris base, 192 mM glycine, and 0.1% SDS at pH 8.3 (Tris-glycine SDS-PAGE). The fractions with the highest protein concentrations were collected and concentrated by centrifuging them in 4 mL Amicon ultra-concentration tubes with a 10 kDa cut-off at 5000× *g* at 4 °C until they reached a suitable working volume and concentration. The next step in purification was performed using size exclusion chromatography with a Superdex 70 10/300 column Enrich SEC 70 (BioRad, Hercules, CA, USA), which was equilibrated with a gel-filtration buffer containing 50 mM Tris-HCl, 200 mM NaCl, and 5 µM β-mercaptoethanol at pH 7.2. Following this step, the sample was concentrated and desalted with simultaneous buffer exchange into storage buffer (50 mM Tris-HCl, pH 7.2, and 5 µM β-mercaptoethanol) using centrifugation in Amicon ultra-concentration tubes with a 10 kDa cut-off at 5000× *g* at 4 °C. The purified enzyme fraction was then analyzed using 12% Tris-glycine SDS-PAGE. Protein bands were visualized by staining the gel with a solution of 2% Coomassie Brilliant Blue R-250 solved in 20% ethanol, 10% acetic acid and 70% of water. The protein concentration of the enzyme was determined with the Bradford assay, following the procedure recommended by the manufacturer (BioRad).

### 2.5. Cellulase Enzyme Activity

To assess cellulolytic activity, we used a method based on the reduction in sugars by 3,5-dinitrosalicylic acid (DNS), which results in a color change from yellow to brown-red [33]. For the quantification of reducing sugars in the reaction mixture, a calibration curve was generated based on the dependence of absorbance (OD_540_) on glucose concentration. We used a water-soluble carboxymethylcellulose (CMC) preparation as a substrate. The enzyme was diluted to a suitable concentration (20 μL) and mixed with 40 μL of a 2% CMC solution in the appropriate condition (pH and temperature) for 60 min. After incubation, 100 μL of DNS reagent (1%) was added to 100 μL of the cellulase reaction mixture, and the solution was incubated for 10 min at 95 °C. High temperatures are required for the stable formation of a red-brown color. After cooling to room temperature, optical density was measured at 540 nm using a spectrophotometer.

### 2.6. Effect of Temperature, pH, Metal Ions, Chelator on Enzyme Activity and Thermostability of the Protein

To determine the optimum temperature of the purified recombinant enzyme, the reaction mixtures were incubated at different temperatures between 37 °C and 75 °C for 1 h. To survey the impact of pH on enzyme activity, the reaction mixtures were assayed in appropriate buffers with different pH values ranging from 4 to 10. For this purpose, in each reaction mixture, the substrate CMC was prepared in 50 mM of citrate buffer for pH 4.0–6.0, in 50 mM Tris-HCl buffer for pH range 6.0–8.0 and in 50 mM glycine-NaOH puffer for pH 9.0–11.0. The impact of cations on the activity of the purified cellulase was evaluated by incubating the reaction mixture with equivalent quantities of various ionic solutions (KCl, NaCl, CaCl_2_, MgSO_4_ and MnSO_4_) at a final concentration of 10 mM. The effect of chelator was evaluated by adding EDTA to final concentrations 1 and 10 mM. To determine the thermal stability, the cellulase sample was preincubated at 80 °C for a number of time intervals. Then, a substrate was added, and an enzymatic reaction was performed under optimal conditions (pH 8.0 and 55 °C, 60 min). All experiments were performed in at least three independent repeats, each in triplicates with means and standard deviations determined to check for errors and variation.

## 3. Results

### 3.1. Metagenomic Analysis of Biragzang Thermal Well and Identification of cel7465 Candidate Gene

**Figure 1:**
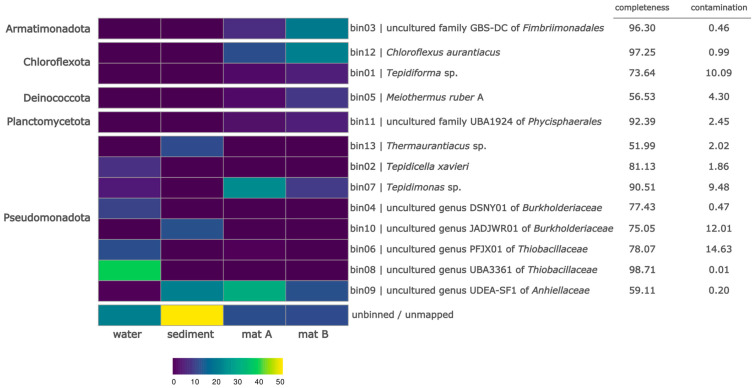
Heatmap of abundance of metagenomic bins derived from shotgun sequencing of samples from Biragzang thermal well. Abundance is given as percentage. The completeness and contamination were determined using CheckM2 software v.1.1.0 [21].

Samples for metagenomic analysis were collected in August 2020 from a deep (2730 m) underground thermal well near the village of Biragzang, located on the right bank of the Ardon River in the Republic of North Ossetia-Alania (Russia). The water exhibited a slightly alkaline reaction, a temperature of 48 °C, and a slightly reducing redox potential. In a previous study, the water, sediments, and microbial mats forming at the well outlet were analyzed using the 16S metabarcoding approach. The analysis revealed that all samples contained a significant number of novel and/or uncultivated microbial taxa (NCBI BioProject accession PRJNA778948). Furthermore, the 16S community profiling indicated that while obligate autotrophic and mixotrophic microorganisms predominate in water, heterotrophs prevail in sediments and microbial mats [34]. In order to investigate the metabolic potential of the Biragzang well, a metagenomic shotgun approach was employed, in which samples of water, sediments, and two samples of microbial mats were subjected to shotgun metagenomic sequencing. A total of 25 million high-quality reads were obtained for the dataset. Following the de novo assembly, binning, and bin refinement procedures, a total of 13 bins were obtained for the dataset (Figure 1). The analysis of bin abundance indicated that approximately 90% of the reads obtained from microbial mats were successfully mapped back to the binned contigs (Figure 1—Bin abundance). This suggests that the obtained bins are representative of the majority of the community. The microbial mats demonstrate a structured community dominated by both cultured and uncultured thermophilic microorganisms, with the most abundant taxon being the uncultured genus UDEA-SF1 (22.65% average abundance), which belongs to the family *Ahniellaceae* within Pseudomonadota—a group typically associated with biofilm formation and metabolic versatility in extreme environments [35] (Figure 1). This was followed by facultative photoautotroph *Chloroflexus aurantiacus* (avg. 17.98%), which utilizes the 3-hydroxypropionate cycle for CO_2_ fixation and oxidizes H_2_S and H_2_ as electron source [36]. The other abundant bins corresponded to *Tepidimonas* sp. (av. 17.29%)—a chemolithoheterotroph specializing in sulfur compound oxidation [37], and an aerobic chemoorganoheterotroph [38] Meiothermus *ruber* (av. 6.06%). Also, we detected a highly abundant representative of *Armatimonadota* phylum (14.35%). This phylum possesses only three valid genera, *Armatimonas*, *Chthonomonas*, and *Fimbriimonas*, which catabolize diverse organic substrates, specializing in proteinaceous and amorphous polysaccharide degradation and encoding numerous carbohydrate-active enzymes [39,40,41]. This knowledge prompted a more thorough examination of the *Armatimonadota* metagenomic bin.

The whole-genome taxonomic analysis of Armatimonadota MAG using GTDB-toolkit [42] classified it as the representative of the uncultured family GBS-DC within *Fimbriimonadales.* This family was identified and characterized through metagenomic analyses across diverse thermal and marine environments. The analysis of GBS-DC metadata in GTDB showed that the family demonstrates remarkable environmental breadth, with representatives recovered from alkaline hot springs in Malaysia (Sungkai, Perak) at elevated temperatures, thermal streams in Uzbekistan [43], and marine macroalgal phycospheres along coastal China [44]. Members of this family have been consistently detected in hot spring sediments in Japan, including the Katase hot spring field at temperatures ranging from 52–84 °C and circumneutral pH conditions [45]. Genomic analyses of GBS-DC-related MAGs, performed by Kato and co-authors, revealed that these bacteria function as aerobic, moderate thermophilic chemoorganoheterotrophs with specialized metabolic capabilities for complex organic carbon degradation [45]. The family’s distribution across both terrestrial geothermal and marine coastal environments indicates versatile adaptation strategies and suggests significant but underexplored contributions to biogeochemical cycling in extreme and marine ecosystems.

**Figure 2:**
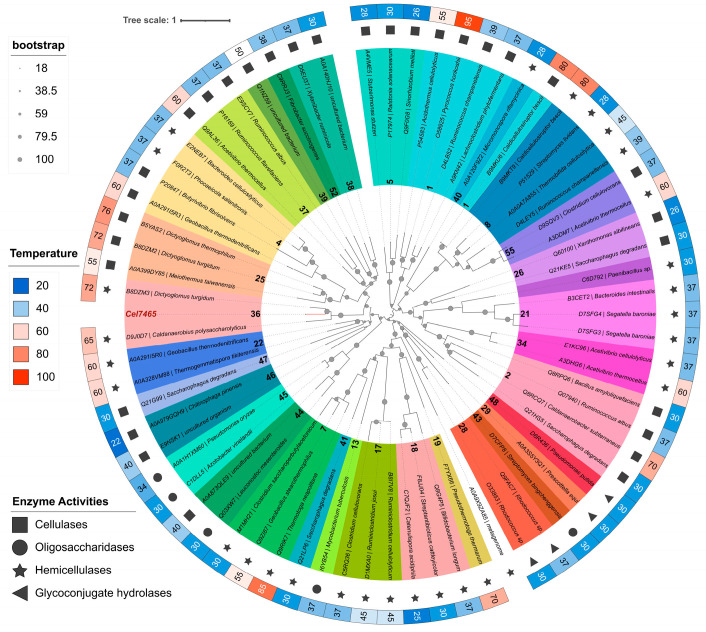
The maximum-likelihood phylogenetic tree of characterized prokaryotic representatives of the glycoside hydrolase family 5 (GH5). The tree was generated with IQ-TREE 3 software. For the visualization purposes the tree was rooted at the midpoint. The bootstrap values are indicated as gray circles. The GH5 subfamilies are highlighted by colors and indicated in the inner circle. The enzyme activities were grouped in four major groups: cellulases included cellulase, exocellulase, endo-1,3-beta-glucanase, glucan 1,3-beta-glucosidase and lichenase; hemicellulases included xyloglucanase, endo-beta-mannanase, beta-mannosidase, xylanase, xylobiase; oligosaccharidases included beta-glucosidase, beta-galactofuranosidase, alpha-arabinosidase and beta-galactosidase; glycoconjugate hydrolases included endoglycoceramidase, galactosylceramidase, beta-N-acetylhexosaminidase. The outer ring represents the heatmap of the optimal growth temperatures of the host of corresponding enzymes. The tree was visualized with iTOL v5 web-server [31].

The annotation of bin03, assigned to GBS-DC family, with NCBI PGAP pipeline [24] revealed the presence of 2621 genes, including 2557 protein-coding genes, 17 pseudogenes and 47 RNA genes. Analysis of in silico proteome of bin03 with dbCAN3 web-server [25] showed the presence of 44 glycoside-hydrolases, only one of which corresponded to potential cellulase belonging to glycoside hydrolase family 5 (GH5), Figure 2, (e-value to GH5.hmm 2.4 × 10^−40^). The phylogenetic analysis of Cel7465 together with biochemically characterized cellulases showed that it falls within the Subgroup 36 of GH5, representing mostly thermostable endoglucanases. To confirm the activity by direct biochemical tests, the gene coding Cel7564 was amplified by PCR and cloned in pET28a vector under control of P_T7_ promoter.

### 3.2. Expression and Purification of the Cel7564 Glycoside Hydrolase

**Figure 3:**
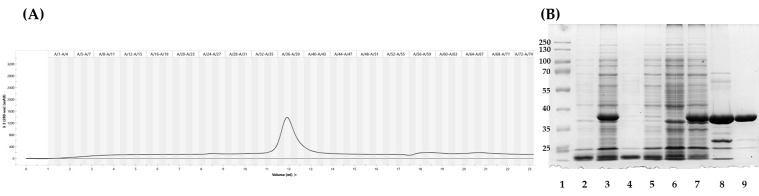
(**A**) Elution profile of the size exclusion chromatography on the Superdex 70 10/300 column. (**B**). Analysis of the expression and purification of recombinant protein Cel7465 with SDS-PAGE (12% polyacrylamide gel) and staining with Coomasie Brilliant Blue R250. Line1: PageRuler™ Plus Prestained Protein Ladder (Thermo Fisher Scientific, Waltham, MA USA); line2: cell lysate of ArcticExpress (DE3) with pET28a vector; line3: cell lysate of ArcticExpress (DE3) with pET28a-cel7465; line4: debris (insoluble proteins) of ArcticExpress (DE3) with pET28a vector; line5: debris (insoluble proteins) of ArcticExpress (DE3) with pET28a-cel7465; line6: cell free extract of ArcticExpress (DE3) with pET28a vector; line7: cell free extract of ArcticExpress (DE3) with pET28a-cel7465; line 8: extract of immobilized metal affinity chromatography (Ni-NTA); line 9: extract of size exclusion chromatography.

The expression of recombinant cellulase Cel7465 in *E. coli* BL21(DE3) pLys and ArcticExpress (DE3) was confirmed using sodium dodecyl sulfate polyacrylamide electrophoresis (SDS-PAGE). A clear band with a molecular weight of approximately 40 kDa was observed. This band corresponded well with the expected molecular weight of the recombinant protein, as predicted from its amino acid sequence. When comparing the specific activity of Cel7465 in cell-free extracts from these two strains, we found that the enzyme activity was almost twice as low in the BL21(DE3) pLys strain, amounting to 156 ± 12 U/mg, compared to the specific activity in the ArcticExpress (DE3) strain, which was 375 ± 27 U/mg. The high activity of the protein in the strain ArcticExpress (DE3) may be due to the correct folding of protein molecules, thus significantly increasing its specific activity. Thus, we chose this strain for further expression and purification of the Cel7465 protein. The enzyme was then purified by IMAC and size exclusion chromatography, the elution profile of size exclusion chromatography is presented on Figure 3A, which demonstrates high level of the protein purification. All the extracts obtained were analyzed using SDS-PAGE, shows that recombinant protein is highly soluble when expressed in *E. coli.* The protein size on the electropherogram (Figure 3B) is close to that predicted by the amino acid sequence.

All purification extracts were tested for the specific enzyme activity. The final activity value, after all purification steps, was approximately 4347 units per milligram of protein (Table 2).

### 3.3. The Effect of the pH and Temperature on the Protein Activity

**Figure 4:**
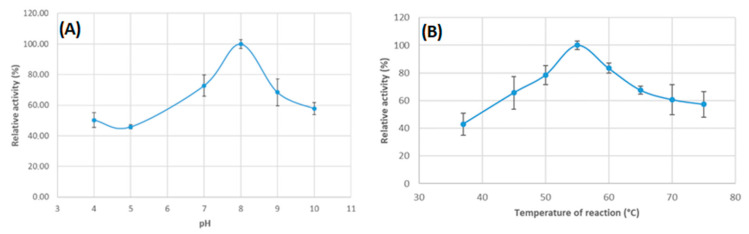
The effects of pH and temperature on the enzymatic activity of recombinant Cel7465. (**A**) Determination of the optimal pH: Assays were conducted at 55 °C for 60 min in buffers with different pH values. Enzyme activity at the optimum pH (8.0) was defined as 100% (**B**) Determination of optimal temperature: Activity was determined in 50 mM Tris-HCl buffer at pH 7.2 at temperatures ranging from 37 °C to 75 °C for 60 min. Enzyme activity at the optimum temperature (55 °C) was defined as 100%.

Experiments to determine the optimal conditions for enzymatic activity, such as reaction temperature and pH, were carried out. The various temperatures (37–75 °C) and pH (4.0–10.0) were tested in order to determine the optimal conditions for the reaction of the recombinant Cel7465 protein. The data are presented in Figure 4A,B. The Cel7465 demonstrated high activity in the pH range from 7 to 9, with an optimal pH of 8.0 (Figure 4A). The enzyme was active over a wide range of pH values, maintaining 50% of its maximum activity at pH 4.0 and remaining active even at pH 9 and 10 (about 60%). Its maximum activity was determined to be 100%, and Cel7465 retained 60% of this activity at high temperatures (60–75 °C). The highest activity was achieved at 55.0 °C (Figure 4B). The results indicates that Cel7465 was active in a wide range of temperatures and pH levels. Nevertheless, we were able to identify the most optimal conditions for the functioning of Cel7465: pH 8.0 and a temperature of 55 °C.

### 3.4. Temperature Stability of the Cel7465 Enzyme

**Figure 5:**
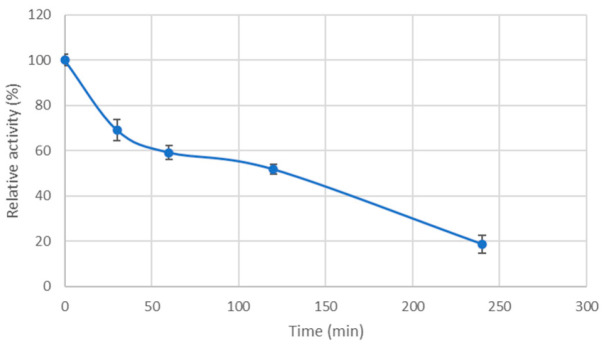
Thermostability of the recombinant Cel7465. Purified enzyme was preincubated at 80 °C for various periods of time before determining the residual activity at 55 °C in 50 mM Tris-HCl buffer (pH 8.0) with CMC as substrate. Enzyme activity without preheating was defined as 100%.

Recombinant Cel7465 maintained approximately 60% of its initial activity after one hour of incubation at 80 °C. After four hours of incubation at the same temperature, the enzyme still retained 20% of its activity (Figure 5). This indicates that the purified protein has a resistance to elevated temperatures during long time of incubation, making it a promising candidate for use in various biotechnology applications.

### 3.5. The Effect of Different Ions on the Reaction Was Also Investigated

Metal ions can bind to proteins and other molecules related to enzymes to form complexes. We tested the effect of various cations on enzyme activity and recorded the reaction of Cel7465 to the tested substances. The results (Table 3) showed that the presence of K^+^ ions inhibit the activity of the enzyme, while Na^+^ ions cause a significant decrease in enzymatic activity. However, divalent ions, such as Mg^2+^ and Ca^2+^, increase the activity of cellulase (to 147.4% and 131.7%, respectively). A significant increase in enzyme activity, more than 7-fold, was observed Mn^2+^ ions were added. The addition of EDTA to the reaction mixture resulted in a loss of enzymatic activity: to 45.6% of the original level at 1 mM and to 22.3% at 10 mM EDTA.

## 4. Discussion

Cellulases are widely used in various industries. It is successfully used for the pulping, bleaching and bioremediation of waste from the pulp and paper industry [46], in the production of biofuels and ethanol [47], and in the textile industry [48,49] for the production of detergents, eliminating roughness on the surface and giving it a smoother appearance [50]. In particular, some cellulases produced by bacteria are used as auxiliary enzymes to help lighten fruit juices [51]. The use of cellulases in the wine and beer industries has also been reported [52,53]. Summarizing all the studies on the use of cellulolytic enzymes, we can conclude that there is a great need to search for, characterize, and obtain new types of cellulases. One of the most promising approaches to finding new glycosyl hydrolases is the use of metagenomes from sources with extreme environmental conditions for microorganisms. In this study, an open reading frame (ORF) was identified during bioinformatics analysis, which was believed to encode the cellulase protein (called Cel7465). A comparison of the amino acid sequence with known sequences in a database (NCBI https://blast.ncbi.nlm.nih.gov, accessed on June 2025) revealed high similarity with the cellulase family (endoglucanase) sequence of a *Fimbriimonadales bacterium* (Sequence ID: MEJ5383462.1). The classification of Cel7465 as an endoglucanase is further supported by its high specific activity toward carboxymethyl cellulose (CMC) that serves as a standard substrate for detecting endoglucanase activity [54].

The new endoglucanase described in this study, Cel7465, represents the first biochemically characterized glycoside hydrolase from the order *Fimbriimonadales*, making it a notable addition to our understanding of enzyme diversity within the phylum Armatimonadota. This phylum, formerly known as candidate division OP10, has remained mostly uncultured despite its widespread distribution in various environments. Only three species have been formally described to date: *Armatimonas rosea*, *Chthonomonas calidirosea*, and *Fimbriimonas ginsengisoli* [39,40,41]. The enzyme originates from the representative of an uncultured GBS-DC family, which has been consistently detected across diverse thermal environments including alkaline hot springs in Japan [45] and Malaysia [55] thermal streams in Uzbekistan [43] and marine hydrothermal systems [56]. Metagenomic analyses of GBS-DC-related genomes suggest their role as aerobic, moderate thermophilic chemoorganoheterotrophs specialized in complex organic carbon degradation [44,45]. The optimal temperature of 55 °C exhibited by Cel7465 aligns well with these genomic predictions and the thermal preferences inferred from environmental distribution patterns. Cel7465 belongs to GH5 subfamily 36, which comprises predominantly uncultured bacterial sequences with demonstrated endo-β-1,4-mannanase and endoglucanase activities [57]. Only three enzymes from this family remain biochemically uncharacterized to date according to the CAZy database [26]. The thermal stability profile of Cel7465, with optimal activity at 55 °C and retention of 60% activity at 80 °C, places it within the moderate thermophile range alongside other characterized GH5 thermostable endoglucanases [58]. This positioning is particularly interesting when compared to other characterized subfamily 36 members. The hyperthermophilic *Dictyoglomus turgidum* DturCelB (Dtur_0671), also from subfamily 36, exhibits high endo-β-1,4-mannanase activity against various mannans but shows lower endoglucanase activity towards CMC, with optimal activity at 70 °C and pH 5.4 [59]. *Caldanaerobius polysaccharolyticus* Man5B, another well-characterized subfamily 36 enzyme, demonstrates clear dual specificity with both mannanase and glucanase activities at optimal temperatures of 60–65 °C [60,61].

The enzyme’s remarkable enhancement of activity (>7-fold increase) in the presence of Mn^2+^ ions represents a unique feature among characterized GH5 endoglucanases [62]. This metal-dependent activation suggests that Cel7465 may have evolved specific binding sites for manganese that stabilize the enzyme conformation or facilitate substrate binding. The inactivation of the enzyme after treatment with the chelating agent (1 and 10 mM EDTA) indicates that the enzyme in its native state contains associated metal ions. Based on the data showing a 7-fold enhancement of activity by Mn^2+^, this ion is the most likely a candidate. Most characterized thermostable mannanases and endoglucanases show moderate enhancement with divalent cations, but few exhibit such dramatic activation [63,64]. The high specific activity of Cel7465 (4347 U/mg after purification) substantially exceeds many reported bacterial endoglucanases, which typically range from 10–1000 U/mg [58,62]. This high catalytic efficiency distinguishes Cel7465 among thermostable carbohydrates and suggests potential advantages for industrial applications where high enzyme turnover is desired. Comparative kinetic studies with other GH5 subfamily 36 members would provide valuable insights into the enzyme’s catalytic mechanism and substrate affinity. The enzyme’s broad pH tolerance, maintaining >50% activity from pH 4.0 to 10.0 with an optimum at pH 8.0, represents an alkaliphilic preference that contrasts notably with DturCelB, which shows optimal activity at the acidic pH of 5.4 [59], most characterized bacterial mannanases exhibit neutral pH optima around 6.0–7.0 [63,65], though some fungal mannanases show acidic optima around pH 4.0 [64]. The environmental origin of Cel7465 from the 48 °C Biragzang thermal well may explain its moderate thermostability profile. This adaptation represents optimization for the specific thermal regime of the source environment rather than extreme thermophily, potentially reflecting evolutionary fine-tuning to local environmental conditions. The combination of high specific activity, moderately alkaline pH preference, and notable thermal stability positions Cel7465 favorably for industrial applications, particularly in processes requiring robust biocatalysts under elevated temperature and pH conditions [66,67,68]. The dramatic enhancement by manganese ions suggests possibilities for process optimization where metal supplementation could significantly boost enzyme performance. The successful metagenomic discovery and heterologous expression of Cel7465 demonstrates the continued value of functional screening approaches for accessing enzymatic diversity from uncultured microorganisms. The Biragzang thermal well environment, with its abundant Armatimonadota representatives (14.35% of the community) and other yet uncultured taxa, suggests significant potential for discovering additional novel enzymes from this underexplored ecosystem. The deep subsurface origin of Cel7465 contributes to growing evidence that such environments harbor remarkable microbial diversity and novel enzymatic capabilities [69]. The enzyme’s properties reflect adaptation to the unique conditions of deep geothermal systems, where elevated temperatures, specific mineral compositions, and isolation from surface influences shape distinct microbial communities.

## 5. Conclusions

The successful metagenomic discovery approach demonstrates the continued potential for accessing enzymatic diversity from uncultured microorganisms in extreme environments, particularly deep subsurface thermal systems that remain largely unexplored. The identification and characterization of Cel7465 makes several important contributions to our understanding of microbial enzyme diversity and biotechnology. As the first biochemically characterized glycoside hydrolase from the order *Fimbriimonadales*, it expands our knowledge of enzymatic capabilities within the understudied phylum Armatimonadota and provides experimental validation of metabolic predictions derived from genomic analysis of the GBS-DC family. The enzyme’s classification within GH5 subfamily 36, known for dual substrate specificity, raises interesting questions about its complete substrate range that warrant further investigation through comprehensive testing against mannan-based substrates. The moderate thermostability of the enzyme, exhibiting optimal activity at 55 °C and retaining significant activity at elevated temperatures, makes it a valuable addition to the repertoire of thermostable biocatalysts. Furthermore, its unique preference for an alkaline pH distinguishes it from most characterized endoglucanases, suggesting promising potential for industrial process optimization. The presence of manganese as a main impurity in the pulp may explain the observed dependence of Cel7465 activity on manganese ions. Consequently, this enzyme is a promising candidate for the efficient hydrolysis of cellulosic biomass.

## Figures and Tables

**Table 1:** Strains and plasmids used in this work.

Strain	Genotype	Reference
*E. coli* DH5a	F^−^ 80Δ*lac*Z M15 (*lac*ZYA-*arg*F) U169 *rec*A1 *end*A1*hsd*R17(rk-, mk+) *pho*A s*upE*44 -*thi*-1 *gyr*A96 *rel*A1	[16], Lab stock
*E. coli* TOP10	F^−^ *mcr*A (*mrr*-*hsd*RMS-*mcr*BC) 80*lac*Z M15 *lac*X74 *rec*A1 *ara* 139 (*ara-leu*)7697 *gal*U *gal*K *rps*L (Str^R^) *end*A1 *nup*G.	Thermo Fischer Scientific, Waltham, CA, USA
*E. coli* BL21(DE3) pLys	F^−^ *ompT hsdS*_B_(r_B_^−^ m_B_^−^) *gal dcm* (DE3) pLysS (Cam^R^)	Novagen
*E. coli* ArcticExpress (DE3)	*E. coli* B (DE3) F *omp*T *hsd*S(rB mB) *dcm*+ Tet^R^*gal end*A Hte [*cpn*10 *cpn*60 Gent^R^]	Agilent Technologies, Santa Clara, CA, USA.
Plasmid	Features	Reference
pUC19	pMB1 replicon *rep* Amp^R^	[17], Lab stock
pET28a	pBR322 origin, f1 origin, T7 promoter, K_M_^R^	Novagen, Burlington, MA, USA
pUC19-cel7465	Gene of the cellulase (*cel*7465) cloned in *Hinc*II restriction site	This work
pET28a-cel7465	Gene of the cellulase (*cel*7465) cloned in restriction site *Bsp*19I and *Sfr*274I	This work

**Table 2:** Purification of recombinant cellulase Cel7465 from *E. coli* strain ArcticExpress (DE3).

Purification Step	Specific Activity (U/mg; µmol/min×mg)	Purification Fold
Cell lysate	761 ± 29	-
Cell free extract	784 ± 36	1.03
Immobilized metal affinity chromatography (Ni-NTA)	2640 ± 139	3.37
Size exclusion chromatography	4347 ± 380	5.7

**Table 3:** The effect of metal ions on enzyme activity. Enzymatic activity without ions was defined as 100%.

Residual Activity of Cellulase (%)	
Control (untreated enzyme)	100 ± 13.8
K^+^	4.9 ± 2.7
Na^+^	45.2 ± 13.3
Mg^2+^	147.4 ± 12.1
Ca^2+^	131.7 ± 11.5
Mn^2+^	786.9 ± 7.9
EDTA 1 mM	45.6 ± 5.6
EDTA 10 mM	22.3 ± 7.5

## Data Availability

The original contributions presented in this study are included in the article/Supplementary Materials. Further inquiries can be directed to the corresponding author. The sequencing and assembly data are submitted to NCBI database under the Bioproject PRJNA778948.

## Supplementary Materials

The following supporting information can be downloaded at: https://www.mdpi.com/article/10.3390/biom15121710/s1, Figure S1: The structure and annotated nucleotide sequence of the recombinant plasmids pUC19-cel7465 (A) and pET28a-cel7465 (B) contain the *cel*7465 gene.
