# Supplementary material for: Cloning and Characterization of the Novel Endoglucanase Identified in Deep Subsurface Thermal Well of Biragzang (North Ossetia) by Metagenomic Analysis
Source: Biomolecules. 2025 Dec 7;15(12):1710. doi: 10.3390/biom15121710 (PMC12731048; doi:10.3390/biom15121710)
Supplement: Supplementary file 1 [file biomolecules-15-01710-s001.zip › biomolecules-4001743-supplementary.pdf]

A)

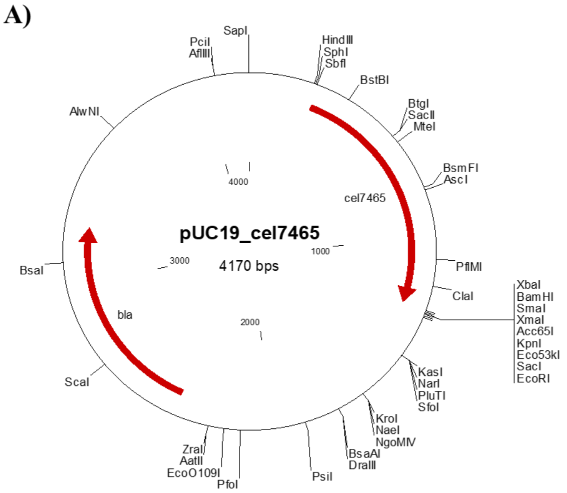

Figure S1. The structure and annotated nucleotide sequence of the recombinant plasmids pUC19-cel7465 (A) and pET28a-cel7465 (B) contain the cel7465 gene.

```
pUC19-cel7465          4170 bp    DNA
FEATURES               Location/Qualifiers
     source            1..4170
                       /mol_type="other DNA"
                       /organism="recombinant plasmid"
                       /SECDrawAs="Info only"
                       /SECName="source"
     CDS               217..254
                       /codon_start=1
                       /gene="lacZ fragment"
                       /product="LacZ-alpha fragment of beta-galactosidase"
                       /label=lacZ-alpha
                       /translation="MTMITPSLHACR"
                       /SECDrawAs="Gene"
                       /SECStyleId=1
                       /SECName="lacZ fragment"
                       /SECDescr="LacZ-alpha fragment of beta-galactosidase"
     misc_feature      234..254
                       /label=MCS
                       /note="pUC18/19 multiple cloning site"
                       /SECDrawAs="Region"
                       /SECStyleId=1
                       /SECName="MCS"
                       /SECDescr="pUC18/19 multiple cloning site"
     CDS               255..1262
                       /codon_start=1
                       /label=cel7465
     misc_feature      1263..1298
                       /label=MCS
                       /note="pUC18/19 multiple cloning site"
     CDS               1263..1551
                       /codon_start=1
                       /gene="lacZ fragment"
                       /product="LacZ-alpha fragment of beta-galactosidase"
                       /label=lacZ-alpha
     CDS               2370..3230
                       /codon_start=1
                       /gene="bla"
                       /product="beta-lactamase"
                       /label=AmpR
                       /note="confers resistance to ampicillin, carbenicillin,
                       and related antibiotics"
     misc_feature      2730..2732
```

```
                     /label=Feature 12
                     /SECDrawAs="Region"
                     /SECStyleId=1
                     /SECName="Feature 12"
ORIGIN
        1 agcgcccaat acgcaaaccg cctctccccg cgcgttggcc gattcattaa tgcagctggc
       61 acgacaggtt tcccgactgg aaagcgggca gtgagcgcaa cgcaattaat gtgagttagc
      121 tcactcatta ggcaccccag gctttacact ttatgcttcc ggctcgtatg ttgtgtggaa
      181 ttgtgagcgg ataacaattt cacacaggaa acagctatga ccatgattac gccaagcttg
      241 catgcctgca ggtcatgacc caacgcacgc tgccggagcc gaccccgcgc aaactcccgc
      301 gctggcgcgg gttcaacctg ctcaataagt tcgggctgga atggagcaac tcgcccttcg
      361 aggagaaaga cttcgaatgg attgccgagc tgggtttcaa cttcgtgcgc ctgccgctgg
      421 actaccgaat ctggaccgag cgcgacaacc cctaccgtct caacgagtcg gagctgcgtg
      481 agattgaccg cgccgtgcag ttcggcgaga agtacgggat tcatgtgcaa ctcaacttcc
      541 atcgcgcgcc gggctacaca gtcgcctcgc ctccagagcc gcggaacctc tggaaagacg
      601 aggaggcgca gcgcatctgc atccatcact ggacgcagtt cgcccgccgc tacaagggca
      661 agccgaaccg ccagctgagt ttcaacctgt tcaacgagcc tgcgaatgtg gacgccgagt
      721 cgcaccgcaa ggtcgtcgag cgcgtggtgg aggcgattcg caaagaggac gctaaccgcc
      781 tgattgtgtg cgacgggcgg gactggggcg gcgcgccgaa cgaagacctg attccgctgc
      841 aggtcgcgca ggcgacgcgc ggctaccagc cgttccgcct cacccactac cgcgccgagt
      901 gggtgcaggg ctccgaccgc tgggagccgc ccaccgaata tcccctgcgc gagggcgagg
      961 tggtgtggga taaaacgcgc ctgtgggagg gctactacgc ccgctggaag acgctggaac
     1021 agaagggcgt cggcgtgatg gtcggcgagt tcggcgcgta ccgacacacg ccccataaag
     1081 tggtgctggc gtggatgcgc gacctgctgg aactgtggaa gcaggcgggc tggggctggg
     1141 cgctgtggaa ctttcgcggc tcgttcggcg tgatcgatag cgagcgcgcc gatgtggcgt
     1201 atcaatcgtg gcgcgggcac aaactggatc gccagatgtt agacctgctg caggcgatgt
     1261 aggactctag aggatccccg ggtaccgagc tcgaattcac tggccgtcgt tttacaacgt
     1321 cgtgactggg aaaaccctgg cgttacccaa cttaatcgcc ttgcagcaca tccccctttc
     1381 gccagctggc gtaatagcga agaggcccgc accgatcgcc cttcccaaca gttgcgcagc
     1441 ctgaatggcg aatggcgcct gatgcggtat tttctcctta cgcatctgtg cggtatttca
     1501 caccgcatac gtcaaagcaa ccatagtacg cgccctgtag cggcgcatta agcgcggcgg
     1561 gtgtggtggt tacgcgcagc gtgaccgcta cacttgccag cgccctagcg cccgctcctt
     1621 tcgctttctt cccttccttt ctcgccacgt tcgccggctt tccccgtcaa gctctaaatc
     1681 gggggctccc tttagggttc cgatttagtg ctttacggca cctcgacccc aaaaaacttg
     1741 atttgggtga tggttcacgt agtgggccat cgccctgata gacggttttt cgccctttga
     1801 cgttggagtc cacgttcttt aatagtggac tcttgttcca aactggaaca acactcaacc
     1861 ctatctcggg ctattctttt gatttataag ggattttgcc gatttcggcc tattggttaa
     1921 aaaatgagct gatttaacaa aaatttaacg cgaattttaa caaaatatta acgtttacaa
     1981 ttttatggtg cactctcagt acaatctgct ctgatgccgc atagttaagc cagccccgac
     2041 acccgccaac acccgctgac gcgccctgac gggcttgtct gctcccggca tccgcttaca
     2101 gacaagctgt gaccgtctcc gggagctgca tgtgtcagag gttttcaccg tcatcaccga
     2161 aacgcgcgag acgaaagggc ctcgtgatac gcctattttt ataggttaat gtcatgataa
     2221 taatggtttc ttagacgtca ggtggcactt ttcggggaaa tgtgcgcgga acccctattt
     2281 gtttattttt ctaaatacat tcaaatatgt atccgctcat gagacaataa ccctgataaa
     2341 tgcttcaata atattgaaaa aggaagagta tgagtattca acatttccgt gtcgccctta
     2401 ttcccttttt tgcggcattt tgccttcctg tttttgctca cccagaaacg ctggtgaaag
     2461 taaaagatgc tgaagatcag ttgggtgcac gagtgggtta catcgaactg gatctcaaca
     2521 gcggtaagat ccttgagagt tttcgccccg aagaacgttt tccaatgatg agcactttta
     2581 aagttctgct atgtggcgcg gtattatccc gtattgacgc cgggcaagag caactcggtc
     2641 gccgcataca ctattctcag aatgacttgg ttgagtactc accagtcaca gaaaagcatc
     2701 ttacggatgg catgacagta agagaattat gcagtgctgc cataaccatg agtgataaca
     2761 ctgcggccaa cttacttctg acaacgatcg gaggaccgaa ggagctaacc gcttttttgc
     2821 acaacatggg ggatcatgta actcgccttg atcgttggga accggagctg aatgaagcca
     2881 taccaaacga cgagcgtgac accacgatgc ctgtagcaat ggcaacaacg ttgcgcaaac
     2941 tattaactgg cgaactactt actctagctt cccggcaaca attaatagac tggatggagg
     3001 cggataaagt tgcaggacca cttctgcgct cggcccttcc ggctggctgg tttattgctg
     3061 ataaatctgg agccggtgag cgtgggtctc gcggtatcat tgcagcactg gggccagatg
     3121 gtaagccctc ccgtatcgta gttatctaca cgacggggag tcaggcaact atggatgaac
     3181 gaaatagaca gatcgctgag ataggtgcct cactgattaa gcattggtaa ctgtcagacc
     3241 aagtttactc atatatactt tagattgatt taaaacttca tttttaattt aaaaggatct
     3301 aggtgaagat cctttttgat aatctcatga ccaaaatccc ttaacgtgag ttttcgttcc
     3361 actgagcgtc agaccccgta gaaaagatca aaggatcttc ttgagatcct ttttttctgc
     3421 gcgtaatctg ctgcttgcaa acaaaaaaac caccgctacc agcggtggtt tgtttgccgg
     3481 atcaagagct accaactctt tttccgaagg taactggctt cagcagagcg cagataccaa
     3541 atactgtcct tctagtgtag ccgtagttag gccaccactt caagaactct gtagcaccgc
     3601 ctacatacct cgctctgcta atcctgttac cagtggctgc tgccagtggc gataagtcgt
     3661 gtcttaccgg gttggactca agacgatagt taccggataa ggcgcagcgg tcgggctgaa
     3721 cggggggttc gtgcacacag cccagcttgg agcgaacgac ctacaccgaa ctgagatacc
     3781 tacagcgtga gctatgagaa agcgccacgc ttcccgaagg gagaaaggcg gacaggtatc
     3841 cggtaagcgg cagggtcgga acaggagagc gcacgaggga gcttccaggg ggaaacgcct
     3901 ggtatcttta tagtcctgtc gggtttcgcc acctctgact tgagcgtcga tttttgtgat
```

3961 gctcgtcagg ggggcggagc ctatggaaaa acgccagcaa cgcggccttt ttacggttcc
4021 tggccttttg ctggcctttt gctcacatgt tctttcctgc gttatcccct gattctgtgg
4081 ataaccgtat taccgccttt gagtgagctg ataccgctcg ccgcagccga acgaccgagc
4141 gcagcgagtc agtgagcgag gaagcggaag
//

B)

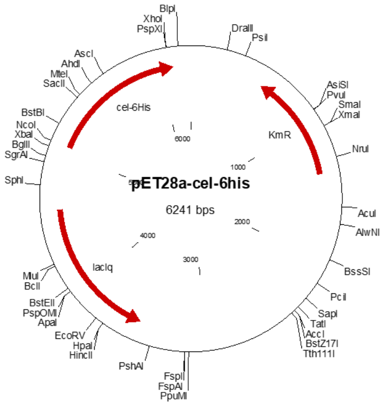

pET28a-cel7465-6his 6241 bp DNA circular
FEATURES Location/Qualifiers
CDS complement(560..1375)
/gene="KmR"
/SECDrawAs="Gene"
/SECStyleId=1
CDS complement(3515..4597)
/gene="lacIq"
/SECDrawAs="Gene"
/SECStyleId=1
misc_feature 4983..5000
/gene="T7-promoter"
/SECDrawAs="Label"
misc_feature 5002..5027
/gene="lac-operator"
/SECDrawAs="Label"
misc_feature 5051..5891
/SECDrawAs="Label"
CDS **5071..6105**
/gene="cel7465-6His"
/SECDrawAs="Gene"
/SECStyleId=1
ORIGIN
1 tggcgaatgg gacgcgccct gtagcggcgc attaagcgcg gcgggtgtgg tggttacgcg
61 cagcgtgacc gctacacttg ccagcgccct agcgcccgct cctttcgctt tcttcccttc
121 ctttctcgcc acgttcgccg gctttccccg tcaagctcta aatcgggggc tccctttagg
181 gttccgattt agtgctttac ggcacctcga ccccaaaaaa cttgattagg gtgatggttc
241 acgtagtggg ccatcgccct gatagacggt ttttcgccct ttgacgttgg agtccacgtt
301 ctttaatagt ggactcttgt tccaaactgg aacaacactc aaccctatct cggtctattc
361 ttttgattta taagggattt tgccgatttc ggcctattgg ttaaaaaatg agctgattta
421 acaaaaattt aacgcgaatt ttaacaaaat attaacgttt acaatttcag gtggcacttt
481 tcggggaaat gtgcgcggaa cccctatttg tttatttttc taaatacatt caaatatgta
541 tccgctcatg aattaattct tagaaaaact catcgagcat caaatgaaac tgcaatttat
601 tcatatcagg attatcaata ccatattttt gaaaaagccg tttctgtaat gaaggagaaa
661 actcaccgag gcagttccat aggatggcaa gatcctggta tcggtctgcg attccgactc
721 gtccaacatc aatacaacct attaatttcc cctcgtcaaa aataaggtta tcaagtgaga
781 aatcaccatg agtgacgact gaatccggtg agaatggcaa aagtttatgc atttctttcc
841 agacttgttc aacaggccag ccattacgct cgtcatcaaa atcactcgca tcaaccaaac
901 cgttattcat tcgtgattgc gcctgagcga gacgaaatac gcgatcgctg ttaaaaggac
961 aattacaaac aggaatcgaa tgcaaccggc gcaggaacac tgccagcgca tcaacaatat
1021 tttcacctga atcaggatat tcttctaata cctggaatgc tgttttcccg gggatcgcag
1081 tggtgagtaa ccatgcatca tcaggagtac ggataaaatg cttgatggtc ggaagaggca
1141 taaattccgt cagccagttt agtctgacca tctcatctgt aacatcattg gcaacgctac
1201 ctttgccatg tttcagaaac aactctggcg catcgggctt cccatacaat cgatagattg
1261 tcgcacctga ttgcccgaca ttatcgcgag cccatttata cccatataaa tcagcatcca
1321 tgttggaatt taatcgcggc ctagagcaag acgtttcccg ttgaatatgg ctcataacac

1381 cccttgtatt actgtttatg taagcagaca gttttattgt tcatgaccaa aatcccttaa
1441 cgtgagtttt cgttccactg agcgtcagac cccgtagaaa agatcaaagg atcttcttga
1501 gatccttttt ttctgcgcgt aatctgctgc ttgcaaacaa aaaaaccacc gctaccagcg
1561 gtggtttgtt tgccggatca agagctacca actctttttc cgaaggtaac tggcttcagc
1621 agagcgcaga taccaaatac tgtccttcta gtgtagccgt agttaggcca ccacttcaag
1681 aactctgtag caccgcctac atacctcgct ctgctaatcc tgttaccagt ggctgctgcc
1741 agtggcgata agtcgtgtct taccgggttg gactcaagac gatagttacc ggataaggcg
1801 cagcggtcgg gctgaacggg gggttcgtgc acacagccca gcttggagcg aacgacctac
1861 accgaactga gatacctaca gcgtgagcta tgagaaagcg ccacgcttcc cgaagggaga
1921 aaggcggaca ggtatccggt aagcggcagg gtcggaacag gagagcgcac gagggagctt
1981 ccagggggaa acgcctggta tctttatagt cctgtcgggt ttcgccacct ctgacttgag
2041 cgtcgatttt tgtgatgctc gtcagggggg cggagcctat ggaaaaacgc cagcaacgcg
2101 gcctttttac ggttcctggc cttttgctgg ccttttgctc acatgttctt tcctgcgtta
2161 tcccctgatt ctgtggataa ccgtattacc gcctttgagt gagctgatac cgctcgccgc
2221 agccgaacga ccgagcgcag cgagtcagtg agcgaggaag cggaagagcg cctgatgcgg
2281 tattttctcc ttacgcatct gtgcggtatt tcacaccgca tatatggtgc actctcagta
2341 caatctgctc tgatgccgca tagttaagcc agtatacact ccgctatcgc tacgtgactg
2401 ggtcatggct gcgccccgac acccgccaac acccgctgac gcgccctgac gggcttgtct
2461 gctcccggca tccgcttaca gacaagctgt gaccgtctcc gggagctgca tgtgtcagag
2521 gttttcaccg tcatcaccga aacgcgcgag gcagctgcgg taaagctcat cagcgtggtc
2581 gtgaagcgat tcacagatgt ctgcctgttc atccgcgtcc agctcgttga gtttctccag
2641 aagcgttaat gtctggcttc tgataaagcg ggccatgtta agggcggttt tttcctgttt
2701 ggtcactgat gcctccgtgt aagggggatt tctgttcatg ggggtaatga taccgatgaa
2761 acgagagagg atgctcacga tacgggttac tgatgatgaa catgcccggt tactggaacg
2821 ttgtgagggt aaacaactgg cggtatggat gcggcgggac cagagaaaaa tcactcaggg
2881 tcaatgccag cgcttcgtta atacagatgt aggtgttcca cagggtagcc agcagcatcc
2941 tgcgatgcag atccggaaca taatggtgca gggcgctgac ttccgcgttt ccagacttta
3001 cgaaacacgg aaaccgaaga ccattcatgt tgttgctcag gtcgcagacg ttttgcagca
3061 gcagtcgctt cacgttcgct cgcgtatcgg tgattcattc tgctaaccag taaggcaacc
3121 ccgccagcct agccgggtcc tcaacgacag gagcacgatc atgcgcaccc gtggggccgc
3181 catgccggcg ataatggcct gcttctcgcc gaaacgtttg gtggcgggac cagtgacgaa
3241 ggcttgagcg agggcgtgca agattccgaa taccgcaagc gacaggccga tcatcgtcgc
3301 gctccagcga aagcggtcct cgccgaaaat gacccagagc gctgccggca cctgtcctac
3361 gagttgcatg ataaagaaga cagtcataag tgcggcgacg atagtcatgc cccgcgccca
3421 ccggaaggag ctgactgggt tgaaggctct caagggcatc ggtcgagatc ccggtgccta
3481 atgagtgagc taacttacat taattgcgtt gcgctcactg cccgctttcc agtcgggaaa
3541 cctgtcgtgc cagctgcatt aatgaatcgg ccaacgcgcg gggagaggcg gtttgcgtat
3601 tgggcgccag ggtggttttt cttttcacca gtgagacggg caacagctga ttgcccttca
3661 ccgcctggcc ctgagagagt tgcagcaagc ggtccacgct ggtttgcccc agcaggcgaa
3721 aatcctgttt gatggtggtt aacggcggga tataacatga gctgtcttcg gtatcgtcgt
3781 atcccactac cgagatatcc gcaccaacgc gcagcccgga ctcggtaatg gcgcgcattg
3841 cgcccagcgc catctgatcg ttggcaacca gcatcgcagt gggaacgatg ccctcattca
3901 gcatttgcat ggtttgttga aaaccggaca tggcactcca gtcgccttcc cgttccgcta
3961 tcggctgaat ttgattgcga gtgagatatt tatgccagcc agccagacgc agacgcgccg
4021 agacagaact taatgggccc gctaacagcg cgatttgctg gtgacccaat gcgaccagat
4081 gctccacgcc cagtcgcgta ccgtcttcat gggagaaaat aatactgttg atgggtgtct
4141 ggtcagagac atcaagaaat aacgccggaa cattagtgca ggcagcttcc acagcaatgg
4201 catcctggtc atccagcgga tagttaatga tcagcccact gacgcgttgc gcgagaagat
4261 tgtgcaccgc cgctttacag gcttcgacgc cgcttcgttc taccatcgac accaccacgc
4321 tggcacccag ttgatcggcg cgagatttaa tcgccgcgac aatttgcgac ggcgcgtgca
4381 gggccagact ggaggtggca acgccaatca gcaacgactg tttgcccgcc agttgttgtg
4441 ccacgcggtt gggaatgtaa ttcagctccg ccatcgccgc ttccactttt tcccgcgttt
4501 tcgcagaaac gtggctggcc tggttcacca cgcgggaaac ggtctgataa gagacaccgg
4561 catactctgc gacatcgtat aacgttactg gtttcacatt caccaccctg aattgactct
4621 cttccgggcg ctatcatgcc ataccgcgaa aggttttgcg ccattcgatg gtgtccggga
4681 tctcgacgct ctcccttatg cgactcctgc attaggaagc agcccagtag taggttgagg
4741 ccgttgagca ccgccgccgc aaggaatggt gcatgcaagg agatggcgcc caacagtccc
4801 ccggccacgg ggcctgccac catacccacg ccgaaacaag cgctcatgag cccgaagtgg
4861 cgagcccgat cttccccatc ggtgatgtcg gcgatatagg cgccagcaac cgcacctgtg
4921 gcgccggtga tgccggccac gatgcgtccg gcgtagagga tcgagatctc gatcccgcga
4981 aattaatacg actcactata ggggaattgt gagcggataa caattcccct ctagaaataa
5041 ttttgtttaa ctttaagaag gagatatacc atggctaccc aacgcacgct gccggagccg
5101 accccgcgca aactcccgcg ctggcgcggg ttcaacctgc tcaataagtt cggcctggaa
5161 tggagcaact cgcccttcga ggagaaagac ttcgaatgga ttgccgagct gggtttcaac
5221 ttcgtgcgcc tgccgctgga ctaccgaatc tggaccgagc gcgacaaccc ctaccgtctc
5281 aacgagtcgg agctgcgtga gattgaccgc gccgtgcagt tcggcgagaa gtacgggatt
5341 catgtgcaac tcaacttcca tcgcgcgccg ggctacacag tcgcctcgcc tccagagccg
5401 cggaacctct ggaaagacga ggaggcgcag cgcatctgca tccatcactg gacgcagttc
5461 gcccgccgct acaagggcaa gccgaaccgc cagctgagtt tcaacctgtt caacgagcct
5521 gcgaatgtgg acgccgagtc gcaccgcaag gtcgtcgagc gcgtggtgga ggcgattcgc
5581 aaagaggacg ctaaccgcct gattgtgtgc gacgggcggg actggggcgg cgcgccgaac

```
     5641 gaagacctga ttccgctgca ggtcgcgcag gcgacgcgcg gctaccagcc gttccgcctc
     5701 acccactacc gcgccgagtg ggtgcagggc tccgaccgct gggagccgcc caccgaatat
     5761 cccctgcgcg agggcgaggt ggtgtgggat aaaacgcgcc tgtgggaggg ctactacgcc
     5821 cgctggaaga cgctggaaca gaagggcgtc ggcgtgatgg tcggcgagtt cggcgcgtac
     5881 cgacacacgc cccataaagt ggtgctggcg tggatgcgcg acctgctgga actgtggaag
     5941 caggcgggct ggggctgggc gctgtggaac tttcgcggct cgttcggcgt gatcgatagc
     6001 gagcgcgccg atgtggcgta tcaatcgtgg cgcgggcaca aactggatcg ccagatgtta
     6061 gacctgctgc aggcgatgct cgagcaccac caccaccacc actgagatcc ggctgctaac
     6121 aaagcccgaa aggaagctga gttggctgct gccaccgctg agcaataact agcataaccc
     6181 cttggggcct ctaaacgggt cttgaggggt tttttgctga aaggaggaac tatatccgga
     6241 t
//
```
